# Nod2 Downregulates TLR2/1 Mediated IL1β Gene Expression in Mouse Peritoneal Macrophages

**DOI:** 10.1371/journal.pone.0027828

**Published:** 2011-11-17

**Authors:** Yogesh Dahiya, Rajeev Kumar Pandey, Ajit Sodhi

**Affiliations:** School of Biotechnology, Faculty of Science, Banaras Hindu University, Varanasi, Uttar Pradesh, India; Charité-University Medicine Berlin, Germany

## Abstract

Nod2 is a cytosolic pattern recognition receptor. It has been implicated in many inflammatory conditions. Its signaling has been suggested to modulate TLR responses in a variety of ways, yet little is known about the mechanistic details of the process. We show in this study that Nod2 knockdown mouse peritoneal macrophages secrete more IL1β than normal macrophages when stimulated with peptidoglycan (PGN). Muramyl dipeptide (MDP, a Nod2 ligand) + PGN co-stimulated macrophages have lower expression of IL1β than PGN (TLR2/1 ligand) stimulated macrophages. MDP co-stimulation have similar effects on Pam3CSK4 (synthetic TLR2/1 ligand) mediated IL1β expression suggesting that MDP mediated down regulating effects are receptor dependent and ligand independent. MDP mediated down regulation was specific for TLR2/1 signaling as MDP does not affect LPS (TLR4 ligand) or zymosan A (TLR2/6 ligand) mediated IL1β expression. Mechanistically, MDP exerts its down regulating effects by lowering PGN/Pam3CSK4 mediated nuclear cRel levels. Lower nuclear cRel level were observed to be because of enhanced transporting back rather than reduced nuclear translocation of cRel in MDP + PGN stimulated macrophages. These results demonstrate that Nod2 and TLR2/1 signaling pathways are independent and do not interact at the level of MAPK or NF-κB activation.

## Introduction

TLRs (toll like receptors) are transmembrane pattern recognition receptors (PRRs) and are among first line of pathogen recognition molecules. Each TLR is now believed to detect a discrete collection of molecules of microbial origin (pathogen/microbe associated molecular patterns, PAMPs/MAMPs) and to signal the presence of infection. TLRs play central role in induction of inflammatory and antimicrobial pathways upon pathogen recognition which ultimately result in immune response and pathogen clearance [1]. Apart from membrane associated PRRs there is another class of PRRs which are cytosolic e.g. NOD (nucleotide binding oligomerization domain) like receptors. Nod1 and Nod2 are most studied representative members of this family [2]. These molecules are believed to detect peptidoglycan degradation products (iE-DAP and MDP being the minimal bioactive motifs respectively) [3], [4]. There are various diseases which show significant association with the mutated forms of cytosolic PRRs. Crohn's disease is such a clinically significant disorder associated with mutant forms of Nod2 [5]. The disease is characterised by chronic intestinal inflammation. There are reports that Nod2 modulates the activity of TLR2 which results in lower Th1 cytokine production after receptor stimulation. In absence of this modulation (e.g. where patients have mutated Nod2) macrophages show enhanced TLR2 mediated Th1 responses which help in progression of disease [6]. On the contrary, there are also reports of synergistic roles of TLRs and Nod2 [7]. But in above cases and in related studies thereafter mechanistic details of Nod2 mediated regulation of TLR signaling are lacking [8]. In this study we asked how Nod2 affects function of TLRs. Is there any preference for a particular TLR and what can be the possible mechanism of action?

We used mouse peritoneal macrophages to show that MDP signaling through Nod2 results in downregulation of PGN (TLR2/1 ligand) induced IL1β expression. However, it was ineffective in downregulating zymosan A (TLR2/6 ligand) or LPS (TLR4 ligand) induced IL1β expression. Our results show that modulatory effects of MDP are restricted to TLR2/1 ligands only. Reduced IL1β expression was observed to be due to reduced levels of cRel in nucleus of MDP + PGN treated macrophages. Reduced levels of cRel in the nucleus of MDP + PGN co-stimulated macrophages are observed to be due to enhanced transporting back from nucleus to cytosol rather than reduced nuclear translocation of cRel.

## Materials and Methods

### Ethics Statement

Studies presented in this manuscript were approved by Scrutiny Committee of School of Biotechnology, Banaras Hindu University, as per University directive no. R/Dev/Project 1987/dt. 31–11–1987.

### Mice

Inbred strains of BALB/c mice of either sex at 8–10 weeks of age were used for obtaining peritoneal macrophages.

### Reagents

MDP, Pam3CSK4 were purchased from Invivogen, San Diego, CA, USA. PGN (*S. aureus*), ultrapure LPS (*E. coli*), zymosan A (*S. cerevisiae*), cycloheximide and most other reagents were purchased from Sigma–Aldrich Chemicals, St.Louis, MO, USA. MAPK inhibitors were purchased from Calbiochem, La Jolla, CA, USA. TLR2 specific neutralizing antibody (clone: T2.5) was purchased from eBiosciences, San Diego, CA, USA.

### Isolation of macrophages and culture conditions

Macrophages were isolated as described previously [9]. Macrophages were cultured in RPMI 1640 medium (Sigma–Aldrich Chemicals, St.Louis, MO, USA) supplemented with 10% fetal calf serum (Biological Industries, Israel), penicillin (100 U/ml), streptomycin (100 U/ml) and gentamycin (20 µg/ml) in a humified CO_2_ incubator. Optimal doses for various stimulants and pharmacological inhibitors were determined by dose kinetics experiments.

### Real Time RT PCR analysis

Total RNA was isolated from the macrophages by TRI reagent (Sigma–Aldrich Chemicals, St.Louis, MO, USA) according to suppliers' instructions. Real time RT-PCR was done using single step real time RT-PCR kit (Qiagen, Hilden, Germany) in Bio-Rad iQ5 real time PCR machine (Bio-Rad Laboratories, Hercules, CA, USA) using SYBR Green detection protocol. Following gene specific primers were used for amplifying genes: IL1β Forward GCAACTGTTCCTGAACTCAACT; Reverse ATCTTTTGGGGTCCGTCAACT; GAPDH Forward TGACCACAGTCCATGCCATC; Reverse GACGGACACATTGGGGGTAG.

Reverse transcription was performed for 30 minutes at 50°C then reverse transcriptase was inactivated at 95°C for 15 min. Amplification was performed with cycling conditions of 94°C for 15 sec, 57°C for 30 sec and 72°C for 30 sec for 35 cycles. After amplification protocol was over, PCR product was subjected to melt curve analysis using Bio-Rad iQ5 software. We used the comparative cycle threshold method (^ΔΔ^C_t_ method) for relative quantitation of gene expression [10]. C_t_ values were calculated by software automatically after completion of run. ^ΔΔ^C_t_ was calculated using formula
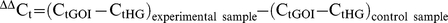



GOI is gene of interest while HG is housekeeping gene.

Fold increase in gene expression was determined using formula 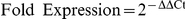



### Measurement of Cytokine production

Concentration of IL1β in culture supernatants and cell lysates was measured by enzyme linked immunosorbant assay using BD OptEIA mouse IL1β ELISA set (BD Biosciences, San Diego, CA, USA) using protocol provided by manufacturer.

Culture supernatants were directly used for secreted IL1β quantification.

### Western Blot Analysis

The macrophage monolayers were washed with ice-cold phosphate-buffered saline containing 1 mM Na_3_VO_4_, lysed in lysis buffer [20 mM Tris-HCl pH 8, 137 mM NaCl, 10% glycerol (v/v), 1% Triton X-100 (v/v), 1 mM Na_3_VO_4_, 2 mM EDTA, 1 mM PMSF, working concentration of mammalian protease inhibitor cocktail] for 10 min at 4°C. The lysates were centrifuged at 15,000 x g for 15 min and the supernatants were separated on 10% SDS-polyacrylamide gels. The separated proteins (40 µg/lane) were transferred to a nitrocellulose membrane (1 hour at 350 mA) using a Bio-Rad Mini Transblotter and membrane was blocked with 5% serum for 2 hours at room temperature and incubated with primary antibody (Santa Cruz biotechnology, Santa Cruz, CA, USA) for 1 hour at room temperature and then with HRP labelled secondary antibody (Santa Cruz biotechnology, Santa Cruz, CA, USA) for 1 hour at room temperature. The blot was developed using ECL reagent (Santa Cruz biotechnology California, USA). To monitor equal loading of protein, western blot analysis using antibody directed against actin was performed.

### Gene knockdown studies

Macrophages were cultured overnight at density of 1x 10^6^ cells per well in 24 well tissue culture plate (Nunc, Roskilde, Denmark). Pre designed siRNA were used for gene knockdown (Santa Cruz, USA). Transfection was performed using N-TER Nanoparticle siRNA Transfection System (Sigma–Aldrich, USA) according to manufacturer instructions. Final siRNA concentration was 100 nM. Transfection was performed for five hours. After five hours medium containing siRNA-complex was removed and replaced by RPMI 1640 supplemented with 10% FCS. Cells were left undisturbed for 48-72 hours after transfection. One set of cells was also transfected with negative control scrambled siRNA to rule out the effects on macrophages because of transfection process. Gene knockdown was confirmed after 24 hours with real time RT-PCR and after 48–72 hours with western blot analysis.

### Electrophoretic Mobility Shift Assay (EMSA)

Nuclear proteins were isolated using NE-PER Nuclear and Cytoplasmic Extraction Reagent kit (Thermo scientific, Rockford, USA). For binding reaction and detection, LightShift Chemiluminiscent EMSA and Chemiluminiscent Nucleic Acid Detection module were used respectively (Thermo scientific, Rockford, USA). Briefly, 100 fmol of labelled probe was incubated with nuclear extract (∼5 µg proteins) in a total volume of 20 µl for 20 min at room temperature with 1× binding buffer. To prevent non-specific binding of nuclear proteins, 100 ng of poly dI:dC was added and the specificity of retarded bands was confirmed by including 100× excess of unlabelled oligonucleotides. Protein-DNA complexes were separated from unbound DNA using 6% native PAGE.

Sequences of probes were as follows: NF-κB probe- 5̀-Biotin-AGTTGAGGGGACTTTCCCAGGC-3̀; AP-1 probe- 5̀-Biotin-CGCTTGATGACTCAGCCGGAA-3̀.

### Statistics

The statistical significance of difference between the test groups was analysed by ANOVA. P value of less than 0.05 was considered significant. The deviation bars of the values represent 95% confidence interval.

## Results

### Stimulation of peritoneal macrophages with PGN but not with MDP triggers IL1β release

**Figure 1:**
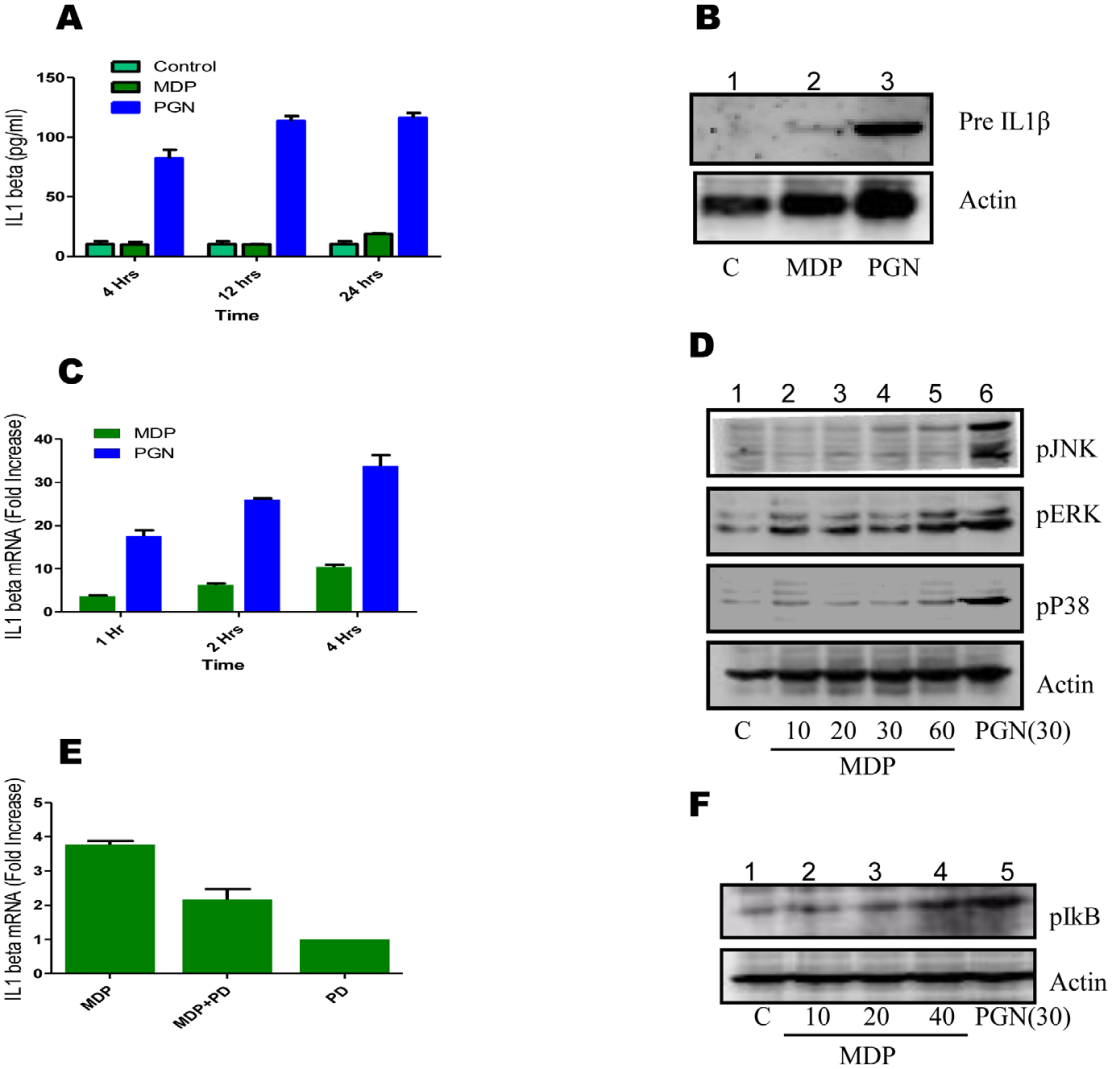
Stimulation of peritoneal macrophages with PGN, but not with MDP, triggers IL1 β **release.** (**A-F**), (**A**) Mouse peritoneal macrophages (1×10^6^/well/ml) were left untreated or treated with MDP (10 µg/ml) or PGN (8 µg/ml) for indicated time intervals. Culture supernatant was checked for the presence of IL1β by ELISA. Statistical significance was checked by two way ANOVA. P value was found to be significant (<0.0001). (**B**) Macrophages were left untreated or treated with MDP or PGN for 4 hours and cell extracts were analysed for the presence of IL1β using western blotting. (**C**) Mouse peritoneal macrophages were left untreated or treated with MDP (10 µg/ml) or PGN (8 µg/ml) for indicated time intervals. Total RNA was isolated using TRI reagent and was checked for the presence of IL1β transcripts using real time RT-PCR. The bars show fold increase compared to control macrophages. Statistical significance was checked by two way ANOVA. P value was found to be significant (<0.0001). (**D**) Macrophages were left untreated or treated as indicated (time in minutes) and cell extracts were analysed for the presence of phosphorylated forms of JNK, ERK and p38 using western blotting. (**E**) Macrophages were pre-treated or left untreated with ERK inhibitor PD98059 (50 µM) for 30 minutes and then stimulated with MDP for 2 hours. Total RNA was isolated and checked for the presence of IL1β transcripts. The graph shows fold increase in IL1β transcripts relative to untreated macrophages. Statistical significance was checked by one way ANOVA. P value was found to be significant (<0.0001). (**F**) Macrophages were left untreated or treated as indicated (time in minutes) and cell extracts were analysed for the presence of phosphorylated forms of IκB using western blotting. Blots correspond to one representative experiment of three independent experiments. Results in graph are presented as the mean of triplicate wells ± SD.

Stimulation of Nod2 has been reported to trigger release of cytokine IL1β [11], [12]. Patients with mutations in CARD15 that makes constitutively active Nod2 are found to release enhanced level of IL1β [13]. Thus, we addressed the question whether Nod2 stimulation with MDP induces IL1β release by mouse peritoneal macrophages. Peritoneal macrophages were treated with MDP (10 µg/ml) or PGN (8 µg/ml). The culture supernatants collected after different time intervals were assayed for presence of secreted IL1β. Only PGN was found to trigger release of IL1β (Fig. 1A).

Intracellular pre-IL1β level was also checked by western blot. It was observed that only PGN stimulated macrophages show enhanced level of pre-IL1β while MDP mediated pre-IL1β level was not significantly changed compared to control macrophages (Fig. 1B).

Transcriptional activation of IL1β gene was also checked by real time RT-PCR. MDP was observed to induce IL1β transcription. The level of IL1β transcripts was significantly lower in MDP treated macrophages as compared to PGN stimulated macrophages (Fig. 1C). To study the molecular mechanism involved in the transcription/expression of IL1β after MDP and PGN stimulation, role of MAPKs was investigated. Only ERK was observed to be activated upon MDP stimulation in peritoneal macrophages while PGN was able to activate p38, JNK and ERK MAPKs (Fig. 1D).

To investigate the role of ERK in MDP mediated IL1β transcription, macrophages were pre-incubated with ERK inhibitor PD98059 (50 µM) for 30 minutes and then stimulated with MDP for 2 hours. Total mRNA was checked for the presence of IL1β transcripts using real time RT-PCR. PD98059 significantly inhibited the MDP mediated transcription of IL1β (Fig. 1E). There are reports of MDP mediated activation of NF-κB [3], [14], [15]. Therefore, MDP mediated NF-κB activation in peritoneal macrophages was investigated. MDP was observed to be a weak stimulant for phosphorylation of IκB as compared to PGN (Fig. 1F).

### PGN mediated IL1β production is through TLR2 and involves NF-κB and JNK pathways

**Figure 2:**
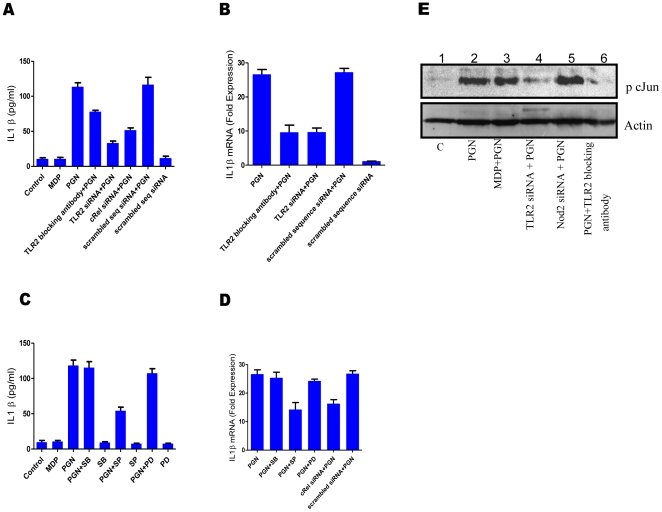
PGN mediated IL1β production is through TLR2 and involves NF-κB and JNK pathways. (**A-E**)**,** Blocking antibody (1 µg/ml) was added 20 minutes prior to stimulation, transfection with gene specific siRNA or scrambled sequence siRNA was done for 72 hours. MAPK inhibitors, SP600125 (20 µM) or PD98059 (50 µM) or SB202190 (5 µM) were added 20 minutes prior to stimulation (**A**) Normal macrophages or TLR2 knockdown macrophages or cRel knockdown macrophages or macrophages pre-treated with TLR2 specific blocking antibody were treated as indicated. After 12 hours culture supernatant was collected and the level of secreted IL1β was measured by ELISA. Each well contained 1×10^6^ macrophages. Statistical significance was checked by one way ANOVA. P value was found to be significant (<0.0001). (**B**) Real time RT-PCR analysis of IL1β mRNA. Normal macrophages or TLR2 knockdown macrophages or macrophages pre-treated with TLR2 specific blocking antibody were treated as indicated. Total RNA was isolated after 2 hours and used for checking IL1β transcripts. Each bar represents fold expression relative to untreated macrophages. Statistical significance was checked by one way ANOVA. P value was found to be significant (<0.0001). (**C**) Macrophages were left untreated or pre-treated MAPK inhibitors (JNK inhibitor SP600125 (20 µM) or ERK inhibitor PD98059 (50 µM) or p38 inhibitor SB202190 (5 µM)) for 30 minutes and then stimulated with MDP or PGN as indicated. Culture supernatants were collected after 12 hours and checked for the presence of secreted IL1β using ELISA. Each well contained 1×10^6^ macrophages. Statistical significance was checked by one way ANOVA. P value was found to be significant (<0.0001). (**D**) Real time RT-PCR analysis of IL1β mRNA. Macrophages were pre-treated with inhibitors or left untreated as described and stimulated with PGN or left unstimulated and checked for the presence of IL1β transcripts after 2 hours of stimulation. Each bar represents fold expression relative to untreated macrophages. Statistical significance was checked by one way ANOVA. P value was found to be significant (<0.0001). (**E**) Western blot analysis of phospho-cJun. TLR2 knockdown macrophages or Nod2 knockdown macrophages or macrophages pre-treated with TLR2 specific blocking antibody were stimulated as indicated. Cell extracts were collected after 40 minutes of stimulation and checked for the presence of phosphorylated form of cJun. Results in graph are presented as the mean of triplicate wells ± SD. Blots correspond to one representative experiment of three independent experiments.

PGN stimulation of macrophages caused transcription and secretion of IL1β. There are contradictory reports on the involvement of TLR2 as receptor for PGN [16]. Therefore, the role of TLR2 in PGN mediated IL1β expression was investigated. Normal or TLR2 knockdown/TLR2 blocking antibody pre-treated peritoneal macrophages were stimulated with PGN. After 12 hours of stimulation culture supernatants were collected and checked for the presence of IL1β. Level of secreted IL1β was found to be significantly lower in TLR2 knockdown/TLR2 blocking antibody pre-treated macrophages as compared to normal macrophages (Fig. 2A). At transcriptional level also similar effects were observed when TLR2 knockdown/TLR2 blocking antibody treated macrophages and normal macrophages were stimulated with PGN for 2 hours and their total RNA was analysed for the presence of IL1β transcripts using real time RT-PCR (Fig. 2B). These observations support the role of TLR2 as PGN receptor.

Involvement of NF-κB in the expression of IL1β has been reported [17]. Therefore, role of NF-κB in PGN mediated IL1β expression in peritoneal macrophages was studied. cRel (a subunit of NF-κB) knockdown macrophages and normal macrophages were stimulated with PGN. Culture supernatants were collected after 12 hours and checked for the presence of IL1β. IL1β level was found to be significantly lower in cRel knockdown macrophages as compared with normal macrophages (Fig. 2A). IL1β transcription on treatment with PGN for 2 hours was also observed to be significantly inhibited in cRel knockdown macrophages (Fig. 2D).

To check the involvement of MAPKs in PGN mediated expression of IL1β macrophages were pre-incubated with JNK inhibitor SP600125 (20 µM) or ERK inhibitor PD98059 (50 µM) or p38 inhibitor SB202190 (5 µM) for 30 minutes and then stimulated with PGN for 2 and 12 hours. PGN mediated IL1β expression was observed to be partially dependent on JNK. Inhibition of ERK and p38 did not affect the expression of PGN mediated IL1β (Fig. 2C, 2D). MAPK activation ultimately leads to the activation of transcription factor AP1 which is a heterodimer of phosphorylated form of cJun and cfos. PGN stimulation of peritoneal macrophages caused phosphorylation of cJun. This phosphorylation was inhibited in TLR2 knockdown macrophages and macrophages pre-treated with TLR2 specific blocking antibody (Fig. 2E).

These observations demonstrate that PGN mediated IL1β expression is through TLR2 and is dependent on JNK and NF-κB pathways in peritoneal macrophages.

### MDP stimulation inhibits PGN mediated IL1β transcription by down regulating NF-κB and AP1 activation

**Figure 3:**
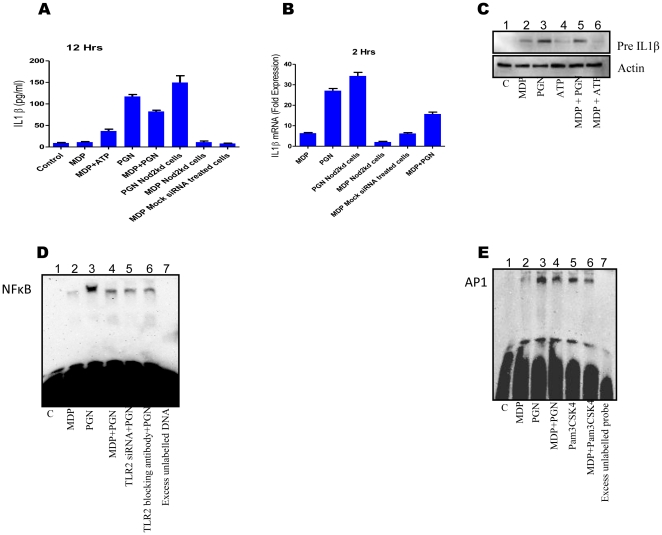
MDP stimulation downregulates PGN mediated IL1β transcription by downregulating NF-κB and AP1 activation. (**A-E**), (**A**) Normal or Nod2 knockdown macrophages were treated as indicated. Culture supernatants were collected after 12 hours and the level of secreted IL1β was measured by ELISA. Each well contained 1×10^6^ macrophages. Statistical significance was checked by one way ANOVA. P value was found to be significant (<0.0001). (**B**) Real time RT-PCR analysis of IL1β mRNA. Normal or Nod2 knockdown Macrophages were treated as indicated. Total RNA was isolated used for checking IL1β transcripts. Each bar represents fold expression relative to untreated macrophages. Statistical significance was checked by one way ANOVA. P value was found to be significant (<0.0001). (**C**) Western blot analysis of pre-IL1β. Peritoneal macrophages were treated as indicated. Cell extracts were collected after 4 hours and analysed for the presence of pre-IL1β. ATP stimulation (1 mM) was given for 20 minutes after primary stimulation (MDP or PGN) was over. Lane1: untreated macrophages (123); lane2: MDP treated macrophages (127); lane3: PGN treated macrophages (140); lane4: ATP stimulated macrophages (124); lane5: MDP+PGN treated macrophages (132); lane6: Macrophages treated with MDP followed by ATP stimulation (108). Values in brackets indicate average integrated density values of spot densitometric analysis using software Alpha Imager (**D**) Electrophoretic mobility shift Assay for NF-κB. Normal or TLR2 knockdown macrophages or macrophages pre-treated (for 20 minutes) with TLR2 specific blocking antibody (1 µg/ml) were stimulated as indicated. Nuclear extracts were prepared and incubated with 100 fmol of biotinylated NF-κB binding sequence. EMSA was performed as described. Lane1: untreated macrophages; lane2: MDP treated macrophages; lane3: PGN treated macrophages; lane4: MDP+PGN treated macrophages; lane5: PGN treated TLR2 knockdown macrophages; lane6: Macrophages treated with TLR2 blocking antibody for 20 min and then stimulated with PGN; lane7: Nuclear extract of PGN stimulated macrophages is incubated with 100 fmol biotinylated NF-κB binding DNA and 100 pmol of unlabelled NF-κB binding DNA. Macrophages were treated for 40 minutes. (**E**) Electrophoretic mobility shift Assay for AP1. Macrophages were treated for 40 minutes as indicated. Nuclear extracts were prepared and EMSA was performed as described. Lane1: untreated macrophages (25); lane2: MDP treated macrophages (45); lane3: PGN treated macrophages (106); lane4: MDP+PGN treated macrophages (87); lane5: Pam3CSK4 treated macrophages (82); lane6: Pam3CSK4+MDP treated macrophages (69); lane7: Nuclear extract of PGN stimulated macrophages is incubated with 100 fmol biotinylated NF-κB binding DNA and 100 pmol of unlabelled AP1 binding DNA (12). Values in brackets indicate average integrated density values of spot densitometric analysis using software Alpha Imager. Western blot and EMSA correspond to one representative experiment of three and two independent experiments respectively.

There are reports for [6] and against [7] MDP mediated down regulation of TLR2 mediated Th1 responses in different systems. To study the effect of any crosstalk between Nod2 and TLR2 signaling, macrophages were treated with MDP, PGN and MDP + PGN. MDP + PGN treated macrophages were found to secrete lesser IL1β (Fig. 3A), had lower IL1β transcripts (Fig. 3B) and had lesser pre-IL1β level (Fig. 3C) when compared with PGN stimulated macrophages. Nod2 knockdown macrophages when stimulated with PGN were found to express more IL1β as compared to normal PGN stimulated macrophages at transcriptional as well as secreted level, while MDP mediated IL1β transcription was found to be significantly less in Nod2 knockdown macrophages as compared to normal macrophages (Fig. 3B). This implies that MDP mediated IL1β transcription is through Nod2 and Nod2 is down-regulating PGN mediated IL1β expression.

As NF-κB and AP1 are involved in PGN mediated IL1β expression, therefore, the effect of MDP on PGN mediated activation of NF-κB and AP1 was investigated by EMSA. PGN mediated NF-κB and AP1 activation was observed to be significantly down-regulated when macrophages were co-incubated with MDP and PGN (Fig. 3D, 3E). AP1 activation by a synthetic TLR2/1 specific ligand Pam3CSK4 was also observed to be down-regulated by MDP co-stimulation (Fig. 3E). This shows that the modulatory effects of MDP are receptor dependent and ligand independent.

### MDP mediated downregulation is specific for TLR2/1 ligands

**Figure 4:**
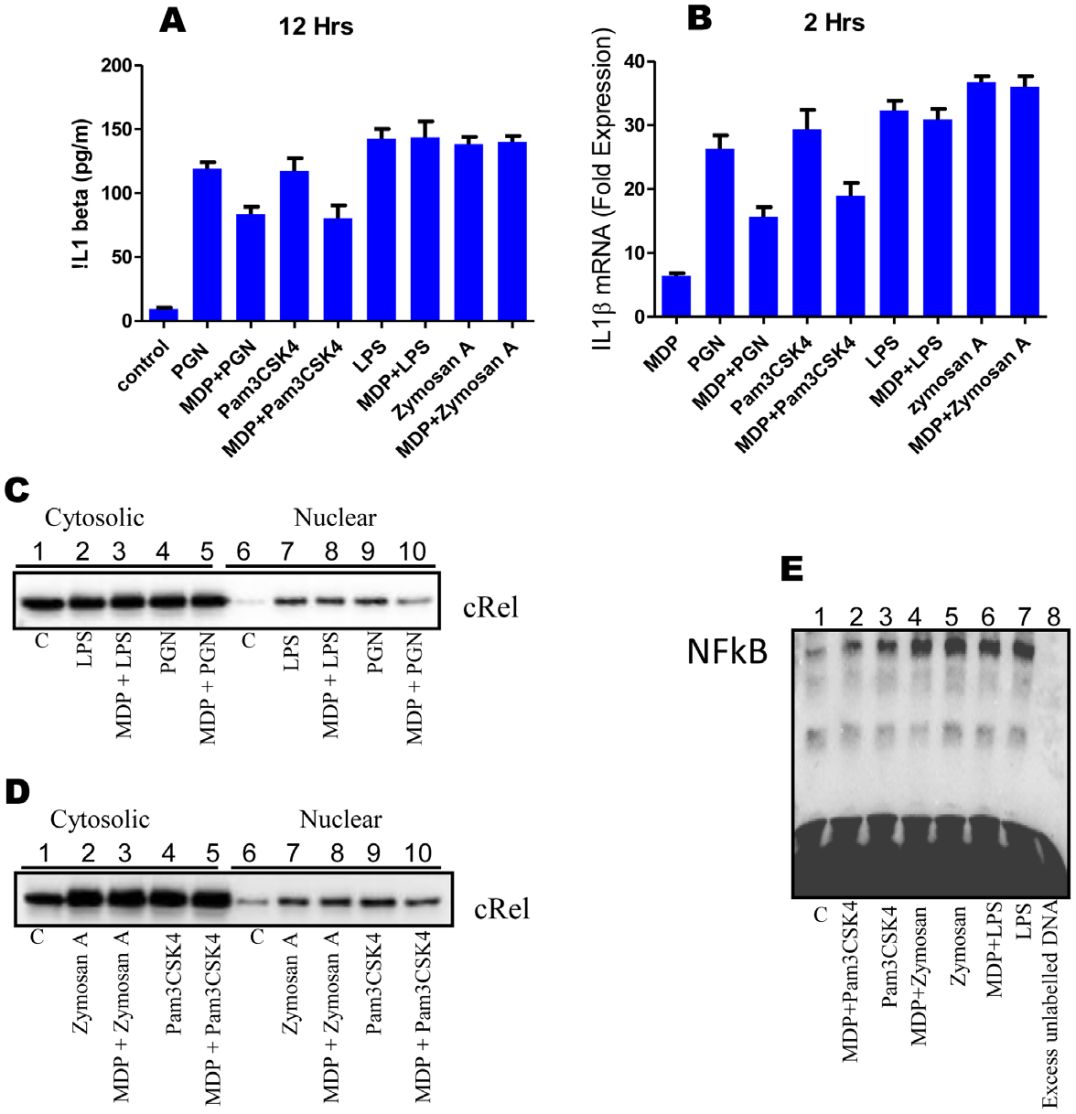
MDP mediated downregulation is specific for TLR2/1 ligands. (**A-E**), (**A**) ELISA for IL1β; macrophages were stimulated as indicated. Culture supernatants were collected after 12 hours and checked for the presence of IL1β. Each well contained 1×10^6^ macrophages. Statistical significance was checked by one way ANOVA. P value was found to be significant (<0.0001). (**B**) Real time RT-PCR analysis of IL1β mRNA: Macrophages were stimulated as indicated for 2 hours. Total RNA was isolated and checked for the presence of IL1β transcripts. Statistical significance was checked by one way ANOVA. P value was found to be significant (<0.0001). (**C and D**) Western blot analyses of nuclear translocation of cRel subunit of NF-κB. Macrophages were stimulated for 40 minutes as indicated. Nuclear and cytosolic extracts were prepared and run on 10% SDS-PAGE. 30 µg of nuclear protein was loaded in each well corresponding to nuclear fraction. (**E**) Electrophoretic mobility shift Assay for NF-κB. Macrophages were stimulated for 40 minutes as indicated. Nuclear extracts were prepared and incubated with 100 fmol of biotinylated NF-κB binding sequence. EMSA was performed as described. Lane1: untreated macrophages (43); lane2: MDP+Pam3CSK4 treated macrophages (59); lane3: Pam3CSK4 treated macrophages (75); lane4: MDP+zymosan A treated macrophages (94); lane5: zymosan A treated macrophages (91); lane6: MDP+LPS treated macrophages (83); lane7: LPS treated macrophages (87); lane8: Nuclear extract of Pam3CSK4 stimulated macrophages is incubated with 100 fmol biotinylated NF-κB binding DNA and 100 pmol of unlabelled NF-κB binding DNA (43). Values in brackets indicate average integrated density values of spot densitometric analysis using software Alpha Imager. Results in graph are presented as the mean of triplicate wells ± SD. Western blot and EMSA correspond to one representative experiment of three and two independent experiments respectively.

To check whether MDP mediated down regulating effects are for all TLRs or they are restricted to TLR2 only, macrophages were co-incubated with MDP and various TLR ligands or with TLR ligands alone. Culture supernatants were collected after 12 hours and analysed for the presence of IL1β by ELISA (Fig. 4A). It was observed that MDP mediated down-regulation of IL1β expression was restricted to PGN and Pam3CSK4 (TLR2/1 ligands) only. LPS (TLR4 ligand) and zymosan A (TLR2/6 ligand [18]) mediated IL1β expression was not affected by MDP co-stimulation. Similar effects were observed at transcriptional level (Fig. 4B).

To investigate whether MDP exerts its TLR2/1 ligand specific down-regulating effects by lowering NF-κB activation, macrophages were stimulated with PGN or MDP + PGN, LPS (1 µg/ml) or MDP + LPS, Pam3CSK4 (5 µg/ml) or MDP + Pam3CSK4, zymosan A (5 µg/ml) or MDP + zymosan A for 40 minutes. Down-regulation in nuclear cRel level (Fig. 4c, 4D) and NF-κB activation (Fig. 4E) was observed in macrophages stimulated with MDP + PGN/Pam3CSK4 compared to PGN/Pam3CSK4 alone treated macrophages while MDP co-stimulation had no effect on LPS and zymosan A mediated cRel nuclear translocation or NF-κB activity. This suggests that observed down-regulating effects of MDP are specific for TLR2/1 receptor.

### MDP co-stimulation facilitate export of nuclear cRel subunits in PGN stimulated macrophages

Lower level of nuclear cRel in MDP + PGN co-stimulated macrophages can be because of either reduced nuclear import (reduced phosphorylation of IκB) or enhanced nuclear export of cRel subunits.

**Figure 5:**
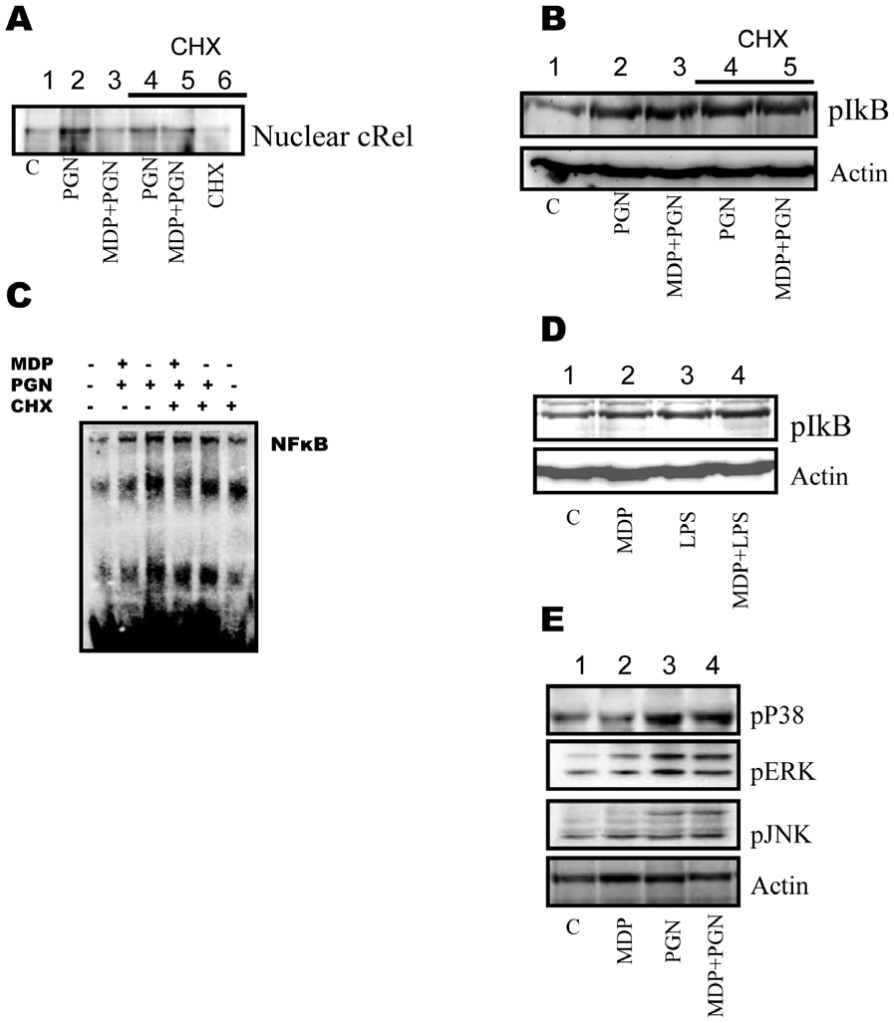
MDP co-stimulation facilitates export of nuclear cRel subunits in PGN stimulated macrophages. (**A-E**), (**A**) Nuclear translocation of cRel. Normal or cycloheximide (5 µg/ml) pre-treated (20 minutes prior to PGN or MDP+PGN stimulus) macrophages were stimulated for 40 minutes as indicated. Nuclear extracts were prepared and run on 10% SDS-PAGE. 30 µg of nuclear extract was loaded per well. Results correspond to one representative experiment of three independent experiments. (**B**) Phosphorylation of IκB. Normal or cycloheximide (5 µg/ml) pre-treated (20 minutes prior to PGN or MDP+PGN stimulus) macrophages were stimulated for 30 minutes as indicated. Whole cell lysates were checked for the presence of phosphorylated form of IκB. Results correspond to one representative experiment of three independent experiments. (**C**) Electrophoretic mobility shift Assay for NF-κB. Macrophages were stimulated for 40 minutes as indicated. Nuclear extracts were prepared and incubated with 100 fmol of biotinylated NF-κB binding sequence. EMSA was performed as described. Cycloheximide treatment was for 20 minutes prior to PGN or MDP+PGN stimulation. Results correspond to one representative experiment of two independent experiments. (**D**) Phosphorylation of IκB. Macrophages were stimulated for 30 minutes as indicated. Whole cell lysates were checked for the presence of phosphorylated form of IκB. Results correspond to one representative experiment of three independent experiments (**E**) Activation of MAPKs. Macrophages were stimulated as indicated for 30 minutes. Cell extracts were analysed for the presence of phosphorylated (activated) forms of p38, ERK and JNK. Results shown correspond to one representative experiment of three independent experiments.

*De novo* synthesis of IκB is negative feedback mechanism for regulation of NF-κB activity. NF-κB stimulating signals usually also lead to synthesis of IκB which has nuclear localization signal. In nucleus it binds to free cRel/p65 and transports them back to cytosol. This ensures transient activation of NF-κB in response to various stimuli [19]. To investigate whether downregulated expression of cRel in nucleus is due to lower nuclear translocation or enhanced transporting back of cRel, macrophages were treated with eukaryotic protein synthesis inhibitor cycloheximide for 20 minutes prior to PGN or MDP + PGN stimulation and checked for the presence of cRel in the nuclear extracts. It was observed that cycloheximide pre-treatment abrogates the capacity of MDP to down-regulate PGN mediated nuclear cRel level (Fig. 5A) and NF-κB activity as checked by EMSA (Fig. 5C). This suggests that MDP mediated signaling is not interfering with pathways that lead to activation of NF-κB (nuclear import of cRel). Surprisingly, there was no significant change in phosphorylation level of IκB in MDP + PGN stimulated macrophages as compared to PGN stimulated macrophages (Fig. 5B). This effect of MDP co-stimulation was found to be same in case of LPS induced IκB phosphorylation (Fig. 5D).

To check whether MDP co-stimulation has any effect on PGN mediated activation of MAPKs (p38, ERK and JNK), macrophages were treated with MDP or PGN or MDP + PGN for 30 minutes and checked for the presence of phosphorylated forms of various MAPKs. PGN mediated activation of p38, ERK and JNK was observed to be unaffected by MDP co-stimulation (Fig. 5E).

## Discussion

Nod2 and TLR signaling affects many immune functions in mice as well as humans. There are reports of interaction of Nod2 signaling with TLR pathways and modulation of those pathways in different ways [2]. But most of the observations are system specific and not all of the functions of TLR2 are affected by Nod2 in the same way. Convincing molecular mechanisms at the early stages of TLR2 and Nod2 interaction are largely unknown. Part of the problem is because of the fact that Nod2 signaling appears to be different in different Nod2 expression cell populations. We found the presence of inflammasome activating agent (ATP) mandatory for MDP to cause secretion of IL1β from peritoneal macrophages (Fig. 3A, 3C) while in some cases Nod2 stimulus alone was sufficient for the effect [12]. Different cell populations also vary in their ability to activate various MAPK in response to MDP [20], [21].

**Figure 6:**
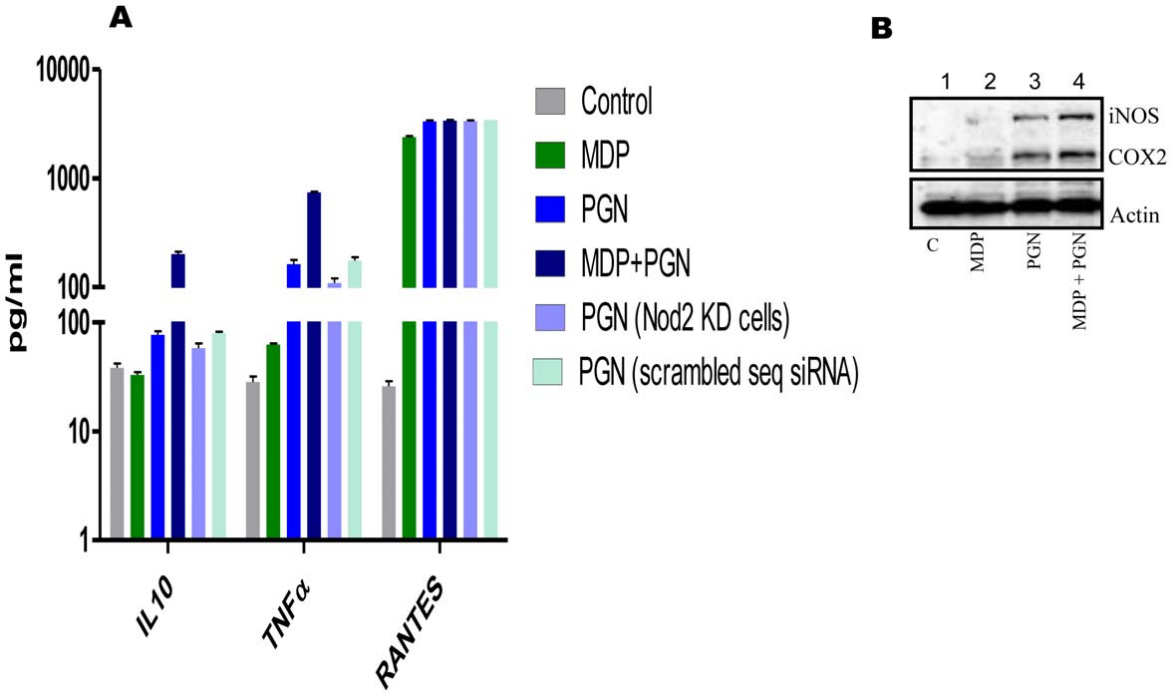
Differential modulation of PGN mediated gene expression by MDP co-stimulation. (**A**) ELISA for IL10, TNFα and RANTES. Normal of Nod2 knockout or scrambled sequence siRNA treated macrophages were stimulated for 12 hours with various ligands as indicated. Culture supernatant was checked for the presence of indicated cytokines. Results are presented as the mean of triplicate wells ± SD. Data is plotted on split semi-log graph. Statistical significance was checked by two way ANOVA. P value was found to be significant (<0.0001). (**B**) Western blot analysis of iNOS and COX2. Macrophages were stimulated for 6 hours as indicated and whole cell lysates were checked for the presence of iNOS and COX2 proteins. Results correspond to one representative experiment of three independent experiments.

Presented data shows that negative regulating effects of MDP are restricted to TLR2/1 ligands only. MDP co-stimulation could interfere with PGN and Pam3CSK4 mediated NF-κB activation, while LPS and zymosan A mediated NF-κB activation remained unaffected. As downstream signaling cascade molecules for NF-κB and MAPK pathways are shared between various TLRs, TLR2/1 specific responses can only be possible when interaction between the TLR2/1 and Nod2 signaling is upstream, may be at the start of the TLR2/1 signaling. In that condition interaction should either weaken or strengthen all TLR2/1 responses but it was clearly not the case. To the contrary, it was observed that MDP mediated Nod2 stimulation does not affect PGN mediated RANTES secretion, while it positively regulates IL10, TNFα, iNOS and COX2 expression (Fig. 6A, 6B). This observation suggests that it is not the universal cytokine expression which is being affected, but it is IL1β (one particular cytokine) expression induced by one particular receptor among many similar types which is being affected. Our results are therefore, different from those obtained by others investigators using splenocytes where authors showed downregulation in cytokine expression induced by PGN as well as LPS [6]. This again shows the cell type specific modulation of TLR activity by Nod2.

Similar levels of cRel/NF-κB activity in nucleus of cycloheximide treated MDP + PGN stimulated macrophages and PGN stimulated macrophages suggests that MDP co-stimulation does not interfere with nuclear import of cRel (Fig. 5A, 5C). Nod2 stimulation was also not found to affect PGN mediated MAPK activation (Fig. 5E). These observations demonstrate that the Nod2 and TLR2/1 pathways may not be interacting at the level of activation of NF-κB and MAPKs. The down-regulating effects observed may be due to the enhanced transporting back of cRel from nucleus to the cytosol in MDP + PGN stimulated macrophages compared to PGN alone stimulated macrophages. A plausible explanation is that there is more unbound NF-κB in the nucleus of MDP + PGN stimulated macrophages which are transported back to the cytosol by the newly synthesized IκB subunits.

**Figure 7:**
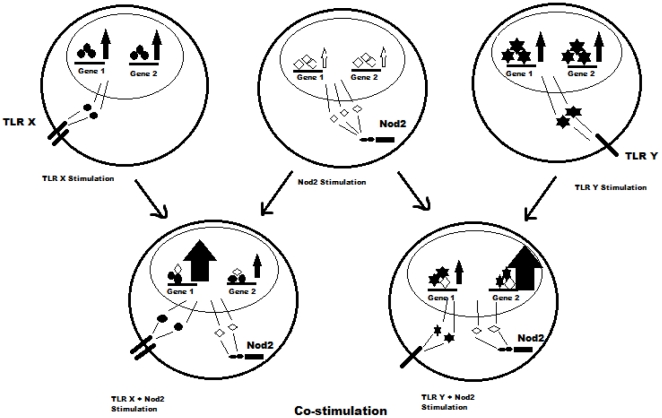
Cartoon diagram showing the effect of Nod2 co-stimulation on gene expression induced by different TLRs. Gene 1 and gene 2 are independently induced by different TLRs and Nod2. Different induction level upon stimulation of different receptors indicates involvement of receptor specific TF profile. Co stimulation of macrophages with MDP and TLR ligands alters the profile of TFs bound to some gene promoters resulting in modulation of expression in various ways.

There are reports of auto activation of Nod2 in systems where it is over expressed [22], [23]. HEK293 cells when transfected with Nod2 expressing plasmid show NF-κB activity even in absence of any stimulus. Nod2 is a molecule that is supposed to be activated by self oligomerization [24], [25] and it is likely that enhanced expression may promote self oligomerization even in absence of ligand. Given the nature of the protein, it is likely that a fraction of the total pool of the protein may be in activated state even in normal physiological concentrations of protein. Here, we propose that macrophages have basal Nod2 activity under unstimulated conditions (i.e. in absence of PAMPs). This activity results in activation of few transcription factors (TFs) with some of them having overlapping binding sites with TFs activated by TLR2/1 signaling. Since each TLR activates a different profile of TFs (as evident by different sets/levels of proteins induced by activation of different TLRs) this mechanism explains for specific regulation of a particular TLR without interfering with its immediate downstream signaling. It assumes that Nod2 do not actually interfere with TLR2/1 signaling, it only affects the activation/function of those few TFs that share a part of binding sequence with the TFs activated by Nod2 or that physically interact with each other at promoter/enhancer elements (Fig. 7). By doing this all the TLR2/1 processes remain intact except a few. As gene expression is dictated by group of activated TFs and profile of activated TFs varies for a given stimulus in different cell types involved, this mechanism can also result in Nod2 dependent positive regulation of some of the processes mediated by TLR2/1 or other TLRs.

This model assumes that Nod2 and TLR2 signaling pathways controlling the expression of IL1β are distinct. They interact only at the level of TF binding to the target sequences. Negative regulation of IL1β is probably a special case. In fact we get positive regulation of iNOS and COX2 genes after MDP co-stimulation as compared to PGN stimulation. Surprisingly, similar signaling pathways are involved in PGN mediated expression of iNOS and COX2 as in the production of IL1β [9]. Our propositions regarding system/receptor specific modulating effects of Nod2 are supported by recent report, which showed synergistic effects of Nod2 and TLR4 on IL1β, iNOS and COX2 expression in RAW264.7 cells [26]. However, they also observed ligand independent functions of over-expressed Nod2 which seems to be its universal (system independent) property.

This mechanism indicates that the interaction of Nod2 and TLRs is complex and relevant only at the system level where Nod2 affects and gets affected by different physiological states. It will be premature to target Nod2 for any therapeutic approaches unless that complex interrelation is better understood.
